# Glyphosate escalates horizontal transfer of conjugative plasmid harboring antibiotic resistance genes

**DOI:** 10.1080/21655979.2020.1862995

**Published:** 2020-12-21

**Authors:** Hongna Zhang, Jingbo Liu, Lei Wang, Zhenzhen Zhai

**Affiliations:** a College of Bioscience and Engineering, Hebei University of Economics and Business, Shijiazhuang City, China; b College of Animal Science and Veterinary Medicine, Shandong Agricultural University, Tai’an City, China; c Shandong Provincial Key Laboratory of Animal Biotechnology and Disease Control and Prevention, Shandong Agricultural University, Tai’an City, China; d Institute of Microbiology, The Second Children & Women’s Healthcare Center of Jinan City, Jinan City, China; e Institute of Microbiology, Tai’an City Central Hospital, Tai’an City, China

**Keywords:** Glyphosate, reactive oxygen species, reactive nitrogen species, antibiotic resistance gene, horizontal transfer

## Abstract

Glyphosate has been frequently detected in water environments because of the wide use for controlling weed in farm lands and urban areas. Presently, the focus of the majority of studies is placed on the toxicity of glyphosate on humans and animals. However, the effects of glyphosate on horizontal transfer of conjugative plasmid carrying antibiotic resistance gene (ARG) are largely unknown. Here, we explored the ability and potential mechanism of glyphosate for accelerating horizontal transfer of conjugative plasmid-mediated ARG. The results showed that glyphosate can effectively boost horizontal transfer rate of conjugative plasmid carrying ARG. The possible mechanism analysis demonstrated that over-production of reactive oxygen species and reactive nitrogen species effectively regulated expression levels of bacterial outer membrane protein and conjugative transfer-related genes, thereby resulting into elevated horizontal transfer rate of plasmid-mediated ARG. In conclusion, this study casts new understanding into the biological effects of glyphosate on ARG.

## GRAPHICAL ABSTRACT

**Figure:**
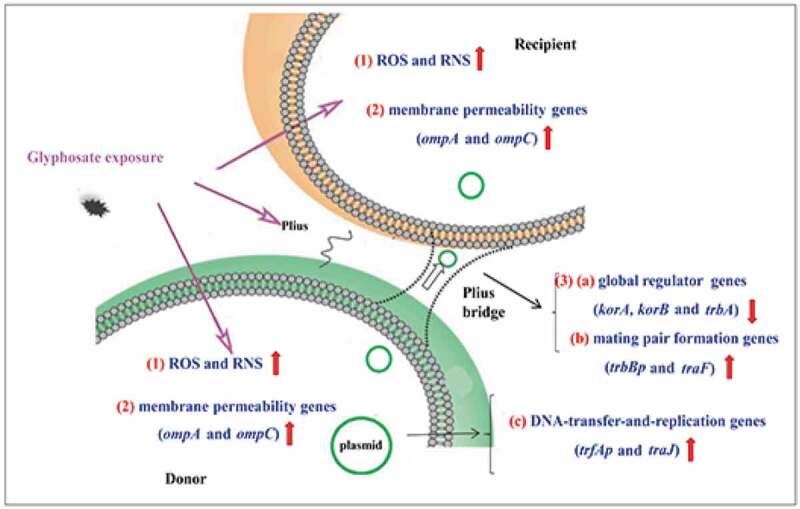

## 1. Introduction

In recent years, a variety of antibiotic resistance genes (ARG) have been frequently detected in different environments, such as water, soil, and air [1,2]. The ever-growing dissemination of ARG has compromised therapeutic efficacy of antibiotics [3,4], therefore increasing health care costs [5,6] and posing a huge threat to the public health [7,8]. The wide use of antibiotics accelerates occurrence and spread of antibiotics resistance pathogens [5,9,10]. The horizontal transfer of conjugative plasmid is a crucial contributor to ARG spread [11,12].

At present, mounting evidence has revealed that besides diverse antibiotics, environmental pollutants, including heavy metals, nanoparticles, disinfectants and non-antibiotic pharmaceuticals, can contribute to ARG transmission [13–18]. It is noteworthy that environmental relevant concentrations of glyphosate can alter bacterial sensitivity to various antibiotics [19].

Glyphosate, the most common active ingredient of herbicides, is widely applied to control weed in farm lands, urban areas, and gardens [20–23]. Presently, glyphosate is frequently detected in various environments, foodstuff and even in the blood and urine of human beings [8,24–29]. Presently, the focus of the majority of studies is placed on the toxicity of glyphosate on humans and animals [30–33], but the effects of glyphosate on horizontal transfer of conjugative plasmid carrying ARG are largely unknown.

To fill the gap, we determined the effect of glyphosate on horizontal transfer rates of conjugative plasmid carrying ARG between *Escherichia coli* (*E. coli*) strains, and then explored the potential molecular mechanism. The results will cast new understanding into the biological effects of glyphosate on ARG spread.

## 2. Materials and methods

### 2.1. Bacteria and glyphosate

The donor bacterium was *E. coli* SD-1 harboring a conjugative plasmid, which has ampicillin (Amp), kanamycin (Kan) and tetracycline (Tet) resistance genes. The *E. coli* SD-2 was the recipient with chloramphenicol (Chl) resistance gene.

Glyphosate (N-[phosphonomethyl]-glycine, >99.7% purity, C3H8NO5P) and four antibiotics (Amp, Chl, Kan and Tet) were bought from Jinan Lvba Bio-Tech CO., LTD (Jinan, Shandong, China).

### 2.2. Influence of glyphosate on bacterial propagation

Luria-Bertani (LB, pH 7.4) broth was used to incubate the donor and recipient bacteria overnight (180 rpm) at 36°C, and then the bacteria were isolated through centrifugation (6 000 rpm) for 5 min. After supernatants were removed, phosphate-buffered saline (PBS, pH 7.2) was used to wash and suspend the pellets. The the donor and recipient bacteria were, respectively, inoculated into LB broth with different environmental-relevant concentrations of glyphosate (0 mg/L, 0.1 mg/L, 0.3 mg/L and 0.6 mg/L) to analyze the growth curve [34]. The selection of glyphosate levels used in this study was based on concentrations of glyphosate in water environments (0.10–0.70 mg/L) [35].

### 2.3. Conjugative transfer rates

After the donor and the recipient bacteria were mixed (1:1 ratio), and the mixture was treated with different concentrations of glyphosate (0 mg/L, 0.1 mg/L, 0.3 mg/L and 0.6 mg/L), respectively [18]. The transconjugants were measured as previously described [14]. In brief, after incubation overnight at 37°C, the mixture (50 μL) were, respectively, plated on LB medium including 4 antibiotics (120.0 mg/L Amp, 40.0 mg/L Kan, 20.5 mg/L Chl and 20 mg/L Tet) for 48 h to count the colonies. The total recipient numbers were determined by LB agar medium containing Chl (20.5 mg/L). The transfer rate was computed by the number of transconjugants to that of the recipients.

### 2.4. Examination of ROS and RNS levels

The production of reactive oxidative species (ROS) and reactive nitrogen species (RNS) was respectively examined using the 2′,7′-Dichlorofluorescein Diacetate (DCFH-DA) (Invitrogen, California, USA) and 4-Amino-5-Methylamino-2′,7′-Difluorofluorescein Diacetate (DAF-FMDA) (Thermo Fisher, Carolina, USA) [36].

### 2.5. Expression levels of genes associated with conjugation

After the mating for 6 h, total RNA was extracted and then cDNA was prepared (TaKaRa, Dalian, China). As previously described [37] (Table 1), quantitative polymerase chain reaction (Q-PCR) was performed to examine expression levels of outer membrane protein (OMP) genes (*ompA* and *ompC*), global regulator genes (*GRG*) (*trbA, korA*, and *korB*), mating pair formation genes (*MPFG*) (*trbBp* and *traF*), and DNA-transfer-and-replication genes (*DTARG*) (*trfAp* and *traJ*).

**Table 1.** Primer sequences of outer membrane protein genes and ones associated with conjugation transfer

Category	Gene	Primer	Sequence of Primer (5ʹ-3ʹ)
Global regulator genes of HGT	*korA*	*korA-F*	TCGGGCAAGTTCTTGTCC
		*korA-R*	GCAGCAGACCATCGAGATA
	*korB*	*korB-F*	CTGGTCGGCTTCGTTGTA
		*korB-R*	TGAAGTCACCCATTTCGGT
	*trbA*	*trbA-F*	TGGAAACTCCCCTACCTCTT
		*trbA-R*	CCACACTGATGCGTTCGTAT
Mating pair formation system genes	*trbBp*	*trbBp-F*	CGCGGTCGCCATCTTCACG
		*trbBp-R*	TGCCCGAGCCAGTACCGCCAATG
	*traF*	*traF-F*	GGCAACCTCGTCGCCTTTA
		*traF-R*	GCAAGTCGGCGTGTTTTCG
DNA transfer and replication system genes	*trfAp*	*trfAp-F*	GAAGCCCATCGCCGTCGCCTGTAG
		*trfAp-R*	GCCGACGATGACGAACTGGTGTGG
	*traJ*	*traJ-F*	GCCCGTGATTTTGTAGCCC
		*traJ-R*	TGAAACCAAGCCAACCAGGAA
Outer membrane protein genes	*ompA*	*ompA-F*	TGAGCCTGGGTGTTTCCTA
		*ompA-R*	CAGAGCAGCCTGACCTTCC
	*ompF*	*ompF-F*	GGTCTGCGTCCGTCCAT
		*ompF-R*	GGTTGCGCCCACTTCA
	*ompC*	*ompC-F*	AAGTAGTAGGTAGCACCAACATCA
		*ompC-R*	GGGCGAACAAAGCACAGAA
16S rRNA	*16S rRNA*	*16s-F*	CCTACGGGAGGCAGCAG
		*16s-R*	ATTACCGCGGCTGCTGG

### 2.6. Statistical analysis

In this study, SPSS 19.0 software was employed to analyze data. Analysis of variance (ANOVA) was used to compare differences among different groups. When *P* values were less than 0.05, the difference denoted significant.

## 3. Results

### 3.1. Conjugative transfer rate and production of ROS and RNS

To truly reflect the influence of glyphosate contamination on antibiotics resistance, the concentrations of glyphosate used in this study followed possible exposure dose human and animal intake from surface water [35]. In addition, the formation of ROS and RNS was examined in the conjugative transfer process.

**Figure 1.**
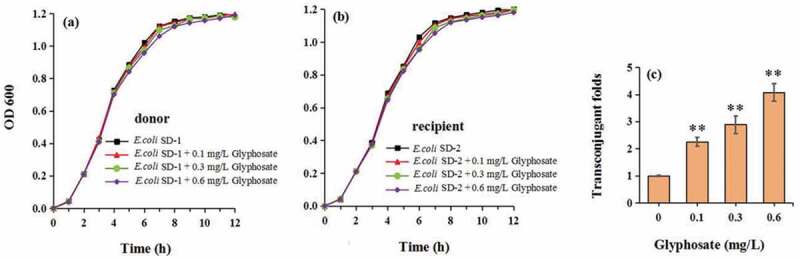
The effect of glyphosate on bacterial propagation (a and b) and conjugative transfer rate of plasmid (c)

**Figure 2.**
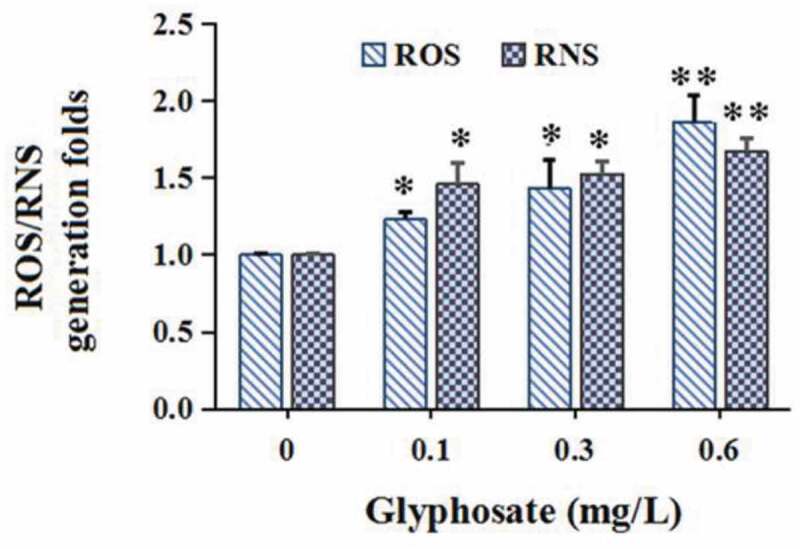
The effect of glyphosate on production of reactive oxygen species (ROS) and reactive nitrogen species (RNS)

Glyphosate concentrations of glyphosate (0.1 mg/L, 0.3 mg/L and 0.6 mg/L) do not affect the propagation of the donor (Figure 1(a)) and recipient strains (Figure 1(b)). Glyphosate exposure definitely increased conjugative transfer rate by 2.26–4.08 times in a concentration-dependant manner (Figure 1(c)). In addition, compared with the control, the ROS and RNS levels were, respectively, increased to 1.23–1.86 and 1.46–1.67 times (Figure 2).

### 3.2. Influence of glyphosate on levels of genes related with conjugation

To explain the possible mechanism of glyphosate for promoting horizontal transfer of plasmid-mediated ARG, we examined expression levels of genes associated with conjugation, including OMP genes, *GRG, MPFG*, and *DTARG*.

**Figure 3.**
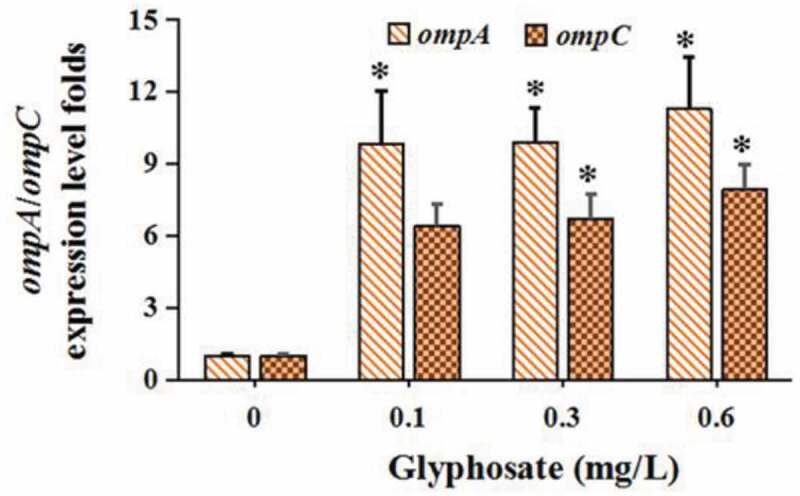
The effect of glyphosate on expression levels of outer membrane protein genes

**Figure 4.**
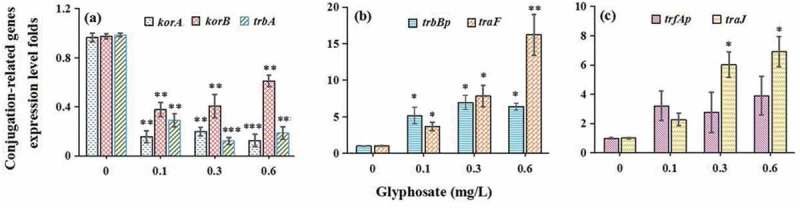
The effects of glyphosate on expression levels of global regulatory genes (a), mating pair formation genes (b) and DNA-transfer-and-replication genes (c)

The levels of *ompA* and *ompC* were, respectively, increased by 9.82–11.26 and 6.38–7.91 folds after the treatment of glyphosate (Figure 3). In terms of genes related to conjugation, we found that the *GRG* (*korA, korB*, and *trbA*) expression levels were suppressed markedly by glyphosate (Figure 4(a)). Two *MPFG* members *trbBp* and *traF* were all up-regulated significantly after the treatment of glyphosate (Figure 4(b)). In addition, glyphosate up-regulated *DTARG* levels (*trfAp* and *traJ*) (Figure 4(c)).

## 4. Discussion

In the current study, the over-formation of ROS and RNS was found, which is in agreement with previous studies to a large extent, which indicated that the over-formation of intracellular ROS or RNS plays a crucial role in accelerating conjugative transfer rate of antibiotic resistance plasmid [18,37–40]. ROS formation can promote conjugative transfer rate of ARG via improving intrachromosomal recombination rates [41]. In addition, nitrite can contribute to peroxynitrite formation, which, as a kind of RNS, can induce SOS response, improving conjugative transfer of ARG [42].

The OMP genes of bacteria, such as *OmpA* and *OmpC* exert a major role in gene transfer and membrane permeability [43–44]. The levels of *ompA* and *ompC* were increased significantly in this study. Many studies have shown that increased OMP expression level can facilitate horizontal transfer of ARG among bacteria via reduced membrane permeability and porins expression levels [,^45–46^].

Conjugation bridge formation between bacteria, an important step for conjugative transfer, is closely related with the *GRG, MPFG* and *DTARG* [47]. In this study, we found that expression levels of three *GRG* members were suppressed markedly. The reduced *korA* and *korB* levels can boost conjugative transmission of antibiotic resistance plasmid between bacteria through up-regulating *trfAp* promoter level [48].

When the mating bridge is formed between bacteria, the MPFG, locating in cell membranes, plays an important role. The bridge can contribute to conjugant formation. In this study, two *MPFG* members *trbBp* and *traF* were all up-regulated significantly after the treatment of glyphosate, which indicated that cell membrane permeability was increased [37].

The *DTARG* plays a crucial role in relaxosome formation and transfer-replication initiation process, therefore contributing to conjugative transfer of plasmid DNA [49]. Glyphosate up-regulated *trfAp* and *traJ* expression levels in this study, which can boost horizontal transfer of plasmid-mediated ARG.

## 5. Conclusion

Taken together, this paper is the first to analyze the effects of glyphosate on conjugative transmission of antibiotic resistance plasmid in water. The result showed that glyphosate can definitely increase conjugative transfer of ARG. In terms of potential mechanisms, cell membrane permeability and conjugative transfer-related genes were regulated via the over-formation of ROS and RNS during bacteria were exposed to glyphosate, therefore increasing the conjugative transfer rates of plasmid-mediated ARG.

## Research highlights

Glyphosate promotes production of ROS and RNS.

ROS and RNS enhance bacterial OMP expression.

ROS and RNS regulate conjugative transfer-related genes.
